# New Findings on the Sperm Structure of Tenebrionoidea (Insecta, Coleoptera)

**DOI:** 10.3390/insects13050485

**Published:** 2022-05-23

**Authors:** Glenda Dias, David Mercati, Paulo Henrique Rezende, José Lino-Neto, Pietro Paolo Fanciulli, Pietro Lupetti, Romano Dallai

**Affiliations:** 1 Departamento de Biologia Geral, Universidade Federal de Viçosa, Viçosa 36570-900, Minas Gerais, Brazil; glendadias1@hotmail.com (G.D.); linoneto@ufv.br (J.L.-N.); 2 Dipartimento Scienze della Vita, Università degli Studi di Siena, Via Aldo Moro 2, 53100 Siena, Italy; david.mercati@unisi.it (D.M.); paolo.fanciulli@unisi.it (P.P.F.); pietro.lupetti@unisi.it (P.L.); 3 Departamento de Entomologia, Universidade Federal de Viçosa, Viçosa 36570-900, Minas Gerais, Brazil; paulo.h.rezende@ufv.br

**Keywords:** insect sperm ultrastructure, basal Tenebrionoidea, sperm looping

## Abstract

**Simple Summary:**

Tenebrionoidea, with more than 30,000 described species and 30 currently recognized families, is a superfamily of difficult taxonomy. The aim of this work is to support the basal position of the Mordellidae among the beetle tenebrionoids. They have a low number of sperm cells per cysts, contrary to the more derived families of the group; moreover, their sperm are not distributed in two bundles at the opposite poles of the cysts, as occurs in the higher taxa, but their sperm flagella form a loop in the median region so that sperm nuclei are positioned close to the tail end. The sperm structure of two members of higher families, Oedemeridae and Tenebrionidae, are investigated to confirm the data mentioned above. The sperm looping, which also occurs in the closely related Ripiphoridae, could be the consequence of the growth asynchrony between the cyst size and the sperm length. The Mordellidae sperm are characterized, not by small mitochondrial derivatives and accessory bodies, but by a peculiar stiff and immotile thin flagellar posterior region provided with only accessory tubules.

**Abstract:**

The sperm ultrastructure of a few representative species of Tenebrionoidea was studied. Two species belong to the Mordellidae (*Mordellistena brevicauda and Hoshihananomia* sp.), one species to Oedemeridae (*Oedemera nobilis*), and one species to Tenebrionidae (*Accanthopus velikensis*). It is confirmed that Mordellidae are characterized by the lowest number of spermatozoa per cyst (up to 64), a number shared with Ripiphoridae. In contrast, in the two other families, up to 512 spermatozoa per cyst are observed, the same number present, for example, in Tenebrionidae. Also, as in the other more derived families of tenebrionoids studied so far, during spermatogenesis in *O. nobilis* and *A. velikensis*, sperm nuclei are regularly distributed in two sets at opposite poles of the cysts. On the contrary, the Mordellidae species do not exhibit this peculiar process. However, during spermiogenesis, the bundles of sperm bend to form a loop in their median region, quite evident in the *Hoshihananomia* sp., characterized by long sperm. This process, which also occurs in Ripiphoridae, probably enables individuals to produce long sperm without an increase in testicular volume. The sperm looping could be a consequence of the asynchronous growth between cyst size and sperm length. The sperm ultrastructure of the Mordellidae species reveals that they can be differentiated from other Tenebrionoidea based on the shape and size of some sperm components, such as the accessory bodies and the mitochondrial derivatives. They also show an uncommon stiff and immotile posterior flagellar region provided with only accessory tubules. These results contribute to a better knowledge of the phylogenetic relationship of the basal families of the large group of Tenebrionoidea.

## 1. Introduction

The Tenebrionoidea constitute one of the largest and most complex superfamilies of beetles [1,2]. A molecular study on the superfamily suggested that it is monophyletic and that four clades have been suggested within the group; among these clades, ripiphorid-mordellid-meloid were considered the most basal in the superfamily [3]. Likewise, Bocak et al. [4], also based on molecular data, considered these three families to be closely related, however in their study the clade formed by them occupies the most derived position in relation to the other Tenebrionoidea. On the other hand, studies by Zhang et al. [5] and McKenna et al. [6], based on extensive gene sampling, maintained Ripiphoridae and Mordellidae as a sister group and in a more basal position of the tenebrionoid tree, while Meloidae appears in a higher position in this tree.

The structure of tenebrionoid sperm is known from Baccetti et al. [7], Dias et al. [8,9], Dallai [10], and Folly et al. [11]. These works have well established that within this group of beetles, the sperm are characterized by a short acrosome, a cylindrical nucleus, and a flagellum with a 9 + 9 + 2 axoneme flanked by two long mitochondrial derivatives and two cylindrical or elliptical accessory bodies [9]. This model, however, is variable in the different families, mainly due to the shape of accessory bodies. In most beetles, testicular sperm bundles, formed at the end of spermatogenesis by cell divisions, contain up to 256 (2^8^) cells. In Tenebrionoidea, this number usually rises to 512 (2^9^), but there are species where this number is only 64 (2^6^), and there are also species where bundles contain 1024 (2^10^) spermatozoa.

Different morphological cellular mechanisms along the spermatogenic process in insects have shown a source of variability in sperm arrangements within cyst cells [12,13]. According to Dias et al. [8], one characteristic shared by members of several families of Tenebrionoidea is that the spermatozoa do not maintain a single orientation within the cyst, as it usually occurs in insects. Nevertheless, during spermiogenesis, their nuclei migrate towards two opposite poles of the cyst, forming two sets of sperm with antiparallel orientation. Also, a unique spermatogenesis mechanism was described for the Hemiptera *Planococcus citri* (Pseudococcidae) [14] and *Kerria chinensis* (Kerriidae) [12]. In this, the result is two sperm bundles separated at the end of spermiogenesis from a process of inverted meiosis, i.e., by a mechanism different from those tenebrionoids described so far. Studying the sperm ultrastructure of some members of three families considered basal [9], it was concluded that Ripiphoridae and Mordellidae have a close phylogenetic relationship. In contrast, Meloidae would be better placed in a more advanced position in the superfamily, as was also suggested, based on molecular data, by Zhang et al. [5] and McKenna et al. [6]. The present work aims to improve our knowledge of tenebrionid sperm structure, extending the study to other group families. In particular, we have examined two new members of Mordellidae, a member of Oedemeridae and one of Tenebrionidae. The results obtained confirm our previous conclusions [9] and give details on a peculiar process, the sperm looping [13], occurring in the testicular cysts in Mordellidae, allowing the sperm to compact in testes of reduced size.

## 2. Materials and Methods

The following species were studied in the present work:

**Mordellidae:**

*Mordellistena (Mordellistena) brevicauda* (Boheman, 1849). Campiglia d’Orcia, Siena, Italy. 12 ex.

*Hoshihananomia* sp. (ex Machairophora sensu Franciscolo, 1943). Viçosa, MG, Brazil. 4 ex.

**Oedemeridae:**

*Oedemera nobilis* (Scopoli, 1763). Gallina, Siena, Italy. numerous ex.

**Tenebrionidae:**

*Accanthopus velikensis* (Piller & Mitterpacher, 1783). Monte Amiata, Siena, Italy. 2 ex.

### 2.1. Light and Epifluorescence Microscopic Preparations

Males of *M. brevicauda*, *O. nobilis* and *A. velikensis* were anesthetized with ether and dissected in 0.1 M phosphate buffer pH 7.2 with 3% of sucrose (PB) to remove the genital system. A drop of sperm, removed from the deferent duct and seminal vesicles, was spread over histological slides and photographed with a Leica DMRB light phase-contrast microscope. The length of spermatozoa was measured using image-J software. For the visualization of sperm nuclei, cells were spread on a histological slide, a drop of 1 ug/mL of the DNA specific dye Hoechst in 0.1 M PB was added, and the sample was finally covered with a glass coverslip. Fluorescence observations of the labelled samples were carried out with a Leica DMRB light microscope equipped with a UV light source, fluorescein, and UV filters and Zeiss AxioCam digital camera with dedicated imaging software.

To observe the entire cysts in *Hoshihananomia* sp., testes were dissected in PB and transferred to a 2% acetic-orcein solution. After 20 min, the follicles were placed on histological slides with a drop of acetic-orcein solution, dissociated using needles, and covered with coverslips. For testicular histology, testes were fixed in 2.5% glutaraldehyde solution in 0.1 M phosphate buffer, postfixed in 1% osmium tetroxide, dehydrated in alcohol solutions, and embedded in Historesin^®^. Semithin sections (0.5 µm thick) were stained with *Giemsa* for 15 min. To measure sperm length, the cells from the vas deferens were spread on histological slides and stained with Giemsa for 15 min. For nuclear size observations and measurements, some samples were stained for 20 min with 0.2 mg/mL DAPI, washed in distilled water, and mounted with 50% sucrose in PB.

### 2.2. Scanning Electron Microscopy (SEM)

Mature spermatozoa taken from the seminal vesicles and deferent ducts of *Mordellistena brevicauda* were spread onto coverslips previously treated with poly-l-lysine. The coverslips were placed in 2.5% glutaraldehyde in PB for 30 min at 4 °C and then rinsed several times in PB. Specimens on glass coverslips were dehydrated in a graded series of ethanol and then processed by critical drying method in a Balzer’s CDP 030. The coverslips were sputtered with about 20 nm gold in a Balzer’s MED 010 sputtering device and finally observed in a SEM Phillips XL20 operating at 10 kV electron accelerating voltage.

### 2.3. Transmission Electron Microscopy (TEM)

Adult males were dissected in PB to isolate the testes and deferent ducts. The material was fixed in 2.5% glutaraldehyde in PB overnight. After careful rinsing, the material was post-fixed in 1% osmium tetroxide for 2 h. After rinsing, the material was dehydrated with ethanol series (50% to 100%), then transferred to propylene oxide, and finally embedded in a mixture of Epon-Araldite resins. Some material was also treated with tannic acid, omitting osmium fixation. Ultrathin sections were obtained with a Reichert Ultracut ultramicrotome, routinely stained with uranyl acetate and lead citrate, and observed with a TEM Philips CM10 operating at 80 kV electron accelerating voltage.

Adult males of the species *Hoshihananomia* sp. were dissected, and the removed testes were processed following the conventional Transmission Electron Microscopy protocol. Ultrathin sections (~60 nm thick) were obtained with an ultramicrotome (Leica UC6). Then they were contrasted with solutions of 3% uranyl acetate and 0.2% lead citrate and after examined in a Transmission Electron Microscope (Tecnai G2-12—SpiritBiotwin FEI) operating at 120 kV at the Microscopy Center of the Federal University of Minas Gerais (CM-UFMG), Belo Horizonte, Minas Gerais, Brazil.

## 3. Results

### 3.1. Mordellistena Brevicauda (Mordellidae)

**Figure 1:**
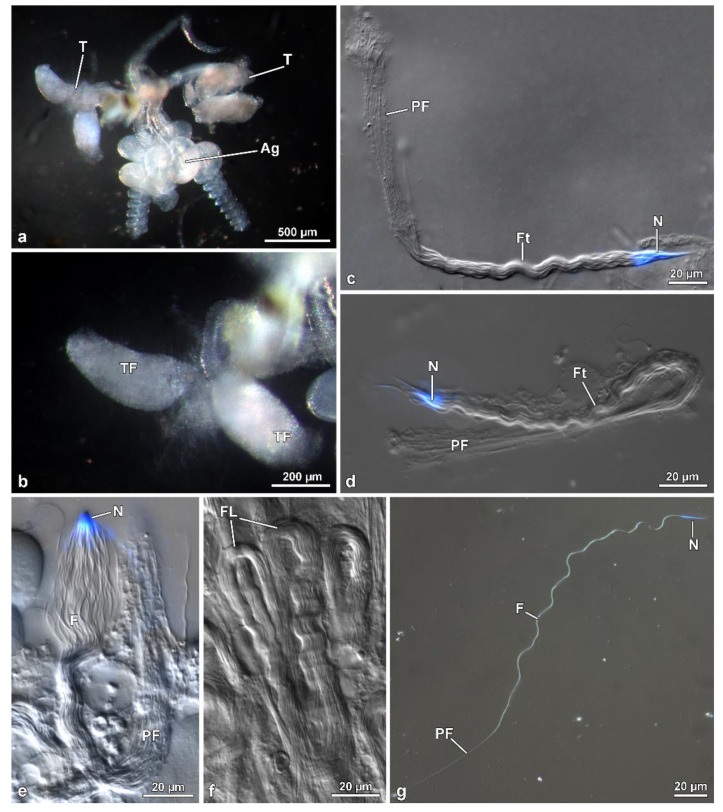
*Mordellistena brevicauda*. Light microscopy of the male genital system organization and the bundles of sperm with looping. (**a**) Male reproductive system with testes (T) and accessory glands (Ag). Note the helicoidal shape of the glandular distal regions. (**b**) Detail of the short, ovoidal testicular follicles (TF). (**c**,**d**) Two sperm bundles after staining with Hoechst showing the nuclei (N) in close contact at only one end. The twisted anterior half (Ft) and, after looping, the rigid and immobile posterior region (PF) are visible. (**e**) A further sperm bundle with the nuclei (N) and the flagella (F), the posterior region of which (PF) are stiff and immotile. (**f**) Some sperm bundles showing the flagella looping (FL). (**g**) Free sperm after Hoechst staining with the apical nucleus (N), the flagellum (F), the posterior region of which is stiff and immotile (PF).

The male reproductive system consists of a pair of testes, each showing 4–5 ovoidal follicles, 480–500 µm long (Figure 1a,b). Testes flow their products into long deferent ducts with large seminal vesicles. They fuse at their proximal end to flow into a long ejaculatory duct. At this level, a complex of spheroidal accessory glands (each 380 µm in diameter) pours their secretions. Two of these glands have a helical long transparent extension, about 600 µm long (Figure 1a). Follicles contain numerous cysts at different stages of spermatogenesis. The elongated ones (Figure 1c–f) consist of maturing spermatids originating from six cycles of cell divisions of a spermatogonium, giving rise to 64 (=26) cells. After Hoechst staining of the long (~260 µm) isolated sperm cysts allowed us to visualize the flagellar ends of the sperms located close to the anterior nuclear regions, all clustered at only one end of the bundle consequent to sperm cells bending by a looping mechanism at about half their length (Figure 1d,e). The anterior region sperm of the bundle shows a twisted appearance, whilst, the posterior tail region is stiff and immotile (Figure 1d–g).

**Figure 2:**
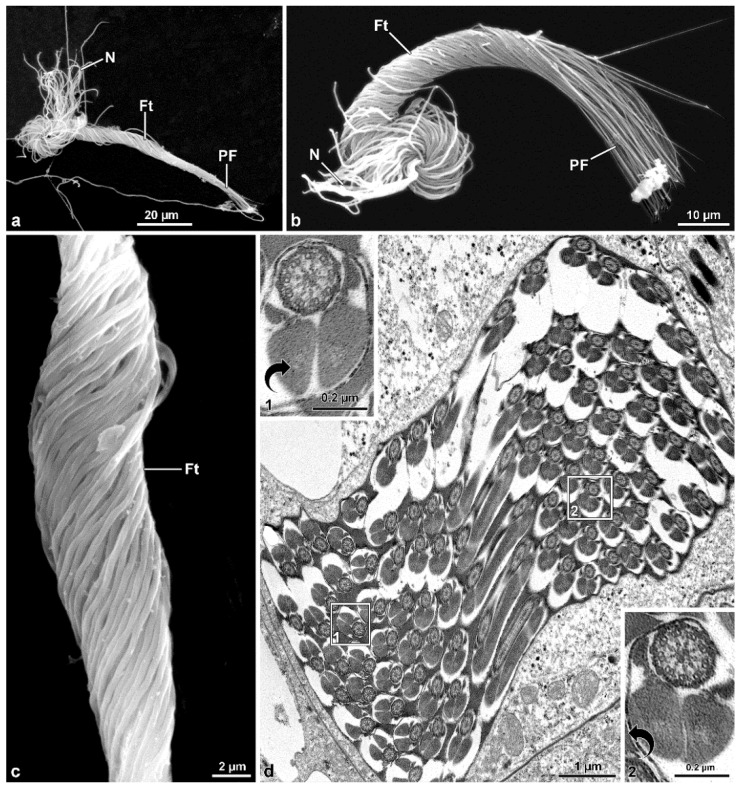
*Mordellistena brevicauda*. The SEM micrographs deal with the sperm flagellum twisting. (**a**,**b**) SEM preparations of two sperm bundles showing the anterior dissociated nuclei (N), the middle twisted flagellar region (Ft), and the posterior stiff and immobile flagellar region (PF). During preparation, the sperm bundle acquires a straight appearance. (**c**) Detail of the twisted flagellar region (Ft) of the sperm bundle. Note the tight array of the sperm flagella. (**d**) Cross-section through a sperm bundle at the base of the loop. The sperm on the upper right side have the doublet microtubules with their dyneins clockwise oriented (see arrow in the inset on the upper left side), while those on the left bottom side have their dynein arms anti-clockwise oriented (see arrow in the inset on the bottom right side). The two sections illustrate the sperm flagella running parallel but in opposite direction.

Scanning electron microscopic preparations confirmed the sperm bundles bending at their half-length; the anterior nuclear region often appears disassembled, the middle regions are tightly twisted, and the posterior regions are thinner and stiff (Figure 2a–c). Sperm cysts are shorter than sperm cells.

**Figure 3:**
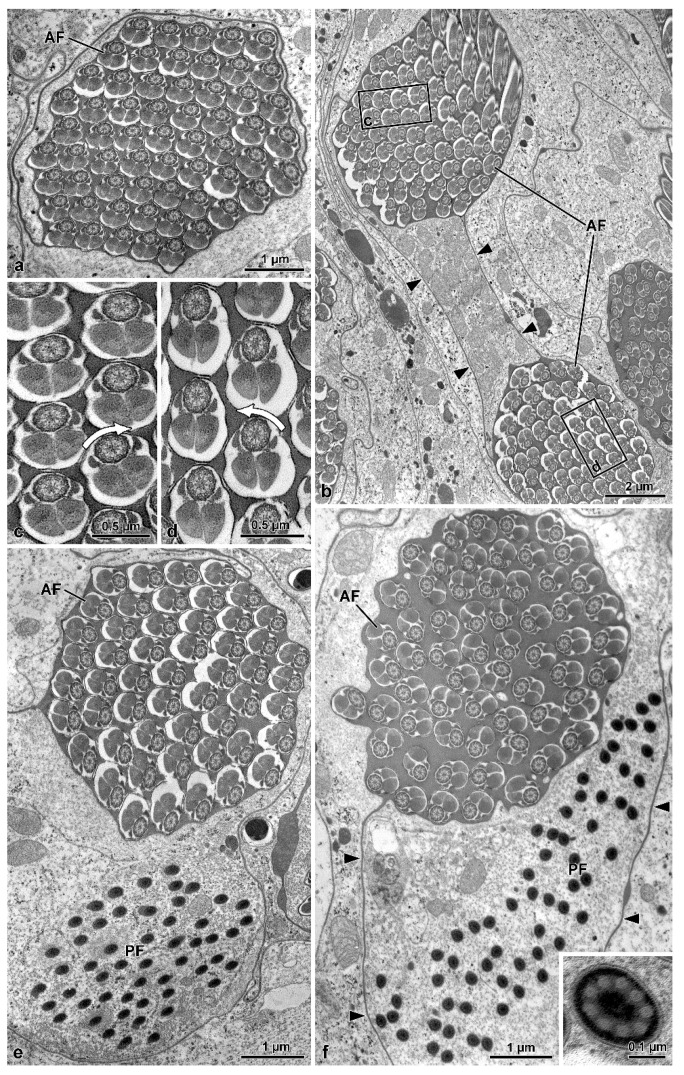
*Mordellistena brevicauda*. The figure illustrates sections of the sperm flagella at different levels indicating the occurrence of sperm looping. (**a**) Cross-section of a sperm bundle with 60 flagella (AF). (**b**) Cross-section of a sperm bundle showing the flagella sectioned at two levels close to the loop. The two flagellar regions (AF) show an opposite orientation of their dynein arms (see (**c**,**d**) with arrows showing dynein arms with opposite orientation). Note also the plasma membrane surrounding the sperm cyst (arrowheads). (**e**,**f**) Two bundles of sperm in cross-sections distant from the loop. Each bundle was sectioned in either the anterior (AF) and the stiff and immotile posterior (PF) flagellar regions. Note the plasma membrane surrounding the sperm cyst (arrowheads). In the inset, a higher magnification of the flagellar axoneme of the stiff and immotile posterior region. Only accessory tubules are visible.

Two antiparallel groups of packed sperm cells are visible in cross-sections cut at about half of the cyst length (Figure 3b), with each group containing from 52 to 61 sperm cells (Figure 3a,e,f). Dynein arms of doublet microtubules of cross-sectioned sperm flagella show opposite orientations between the two sperm groups (Figure 3b–d). All the sperm from one group show the dynein arms clockwise oriented, typical of sperm observed from the centriolar region to the tail. In contrast, the sperm from the contiguous group show the dynein arms anti-clockwise-oriented, as expected for sperm observed from the tail end (Figure 3a–c). The same pattern is also visible when the cross-section is through the loop region (Figure 2d) This antiparallel orientation between the two groups of sperm from the same cyst is due to the sperm looping occurring in the cysts as shown in Figure 1d–f. The plasma membrane of cystic cells enwrapping the two antiparallel groups of spermatozoa is clearly visible (Figure 3b–f). When cysts are cross-sectioned at levels far from the loop region, i.e., near their two opposite ends, sperm flagella of one group show their anterior regions, while sperm flagella of the other group exhibit their thin caudal ends (Figure 3e,f).

**Figure 4:**
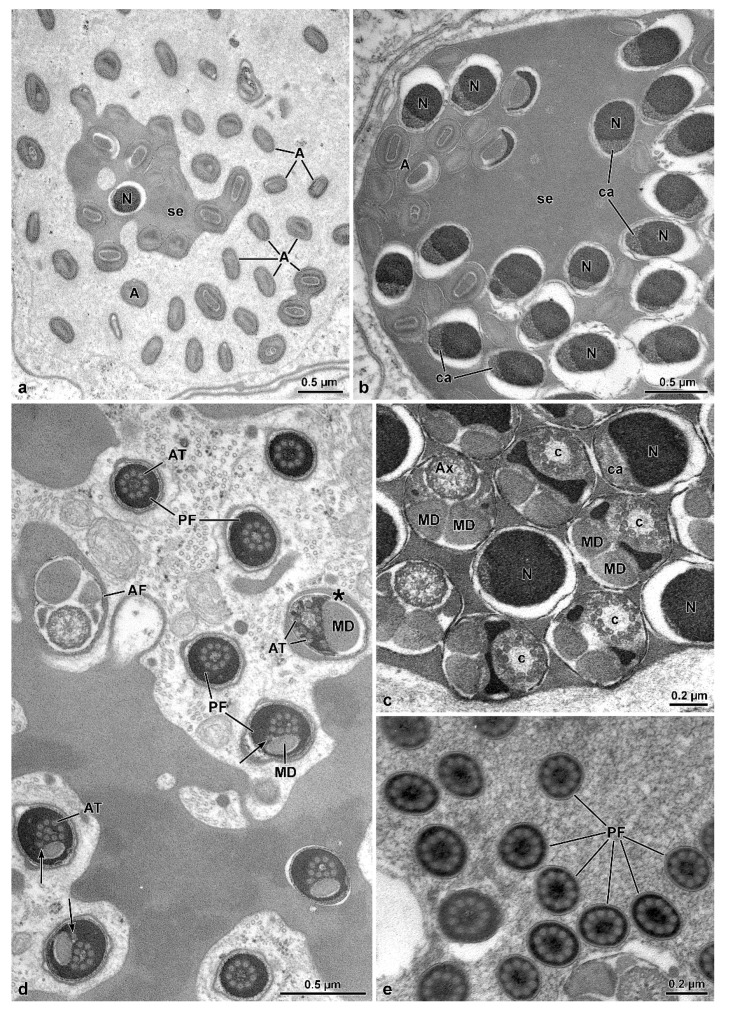
*Mordellistena brevicauda*. The TEM micrographs illustrate the fine structure of the sperm at different levels. (**a**,**b**) Cross-sections through acrosomes (A) and nuclei (N) embedded in an electron-dense secretion (se). ca, centriole adjunct material. (**c**) Cross-section of the transition region between nucleus (N) and flagellum showing centriole (c), centriole adjunct material (ca), axoneme (Ax), and mitochondrial derivatives (MD). (**d**) Cross-section of the transition region between the normal (AF) and the posterior stiff flagella (PF) regions. Residual axoneme is sometimes visible (asterisk) with central and doublet microtubules. More often only accessory tubules (AT) are visible. Mitochondrial derivatives (MD) are greatly reduced or disappeared. Arrows indicate the accessory tubule outside the crown of these tubules. (**e**) Cross-section of the stiff and immotile posterior flagella (PF), in which only accessory tubules are present, embedded in electron-dense material.

The length of the sperm is 290–305 µm, of which 1.3 µm corresponds to a conical acrosome and 15 µm to the nucleus (Figure 1g). The acrosome is slightly elliptical in cross-section, 0.43 µm in diameter, and has a dense perforatorium with the same slightly elliptical shape (Figure 4a,b). The sperm are embedded for almost their whole length in a homogeneous electron-dense material (Figure 3a–f). After sample processing for either light or SEM observations, this material seems to be removed, and the anterior sperm regions appear separated, while the middle regions are still tightly twisted (Figure 2a–c). The nucleus, 0.5–0.3 µm in diameter, is tapered from the base to the apex and contains a compact chromatin (Figure 4a–c). It exhibits a flattened side along its length, which is closely associated with a finely granular material of the centriole adjunct (Figure 4b,c). In cross-section, these two structures together result in an oval profile near the acrosome (Figure 4b) and circular in the basal region (Figure 4d). At the nuclear anterior end, a lateral groove hosts the perforatorium base and, more externally, the asymmetric base of the acrosome (Figure 4b). The posterior nuclear region, on one side, has two cavities hosting the proximal tips of the two mitochondrial derivatives, and on the opposite side, a cavity that houses the centriole (or basal body) (Figure 4c).

The centriole consists of a complex of 9 microtubule doublets, devoid of dynein arms, and of a crown of 9 outer accessory tubules (Figure 4c). A cross-section of this region shows how the different sperm components are integrated, including the narrow nuclear lamina (Figure 4c). Beneath the centriole, the nucleus is no longer visible and all flagellar components are evident: a 9 + 9 + 2 axoneme, two similar mitochondrial derivatives, and two small, almost triangular or elliptical accessory bodies (Figure 4c). Small cisterns are present between the axoneme and the mitochondrial derivatives. Towards the posterior flagellar region, one of the mitochondrial derivatives exhibits a larger diameter and ends proximally than the slimmer one (Figure 4d). The posterior flagellar region, about 100 µm long (Figure 1c–e,g), has a remarkably different organization (Figure 4d,e). In the transition between the above described conventional flagellar structure and the posterior sperm region, the reduction of flagella diameter, from approximately 0.5 µm to 0.3 µm occurs, accompanied by the progressive disappearance of the axoneme components. This region is characterized by a very dense material, in which a single mitochondrial derivative and the altered axoneme are embedded. This latter loses first the peripheral doublet microtubules, then the central pair of tubules, and finally the radial links (Figure 4d). Besides the single mitochondrial derivative, only eight accessory tubules are visible in a circle, while one of them is shifted laterally (Figure 4d). Further posteriorly, when no mitochondria derivatives are visible, the nine accessory tubules take the conventional circular array that continues all the way down to the tail tip (Figure 4e). Due to lack of the axonemal doublets, the entire region has to be immotile.

### 3.2. Hoshihananomia sp. (Mordellidae)

**Figure 5:**
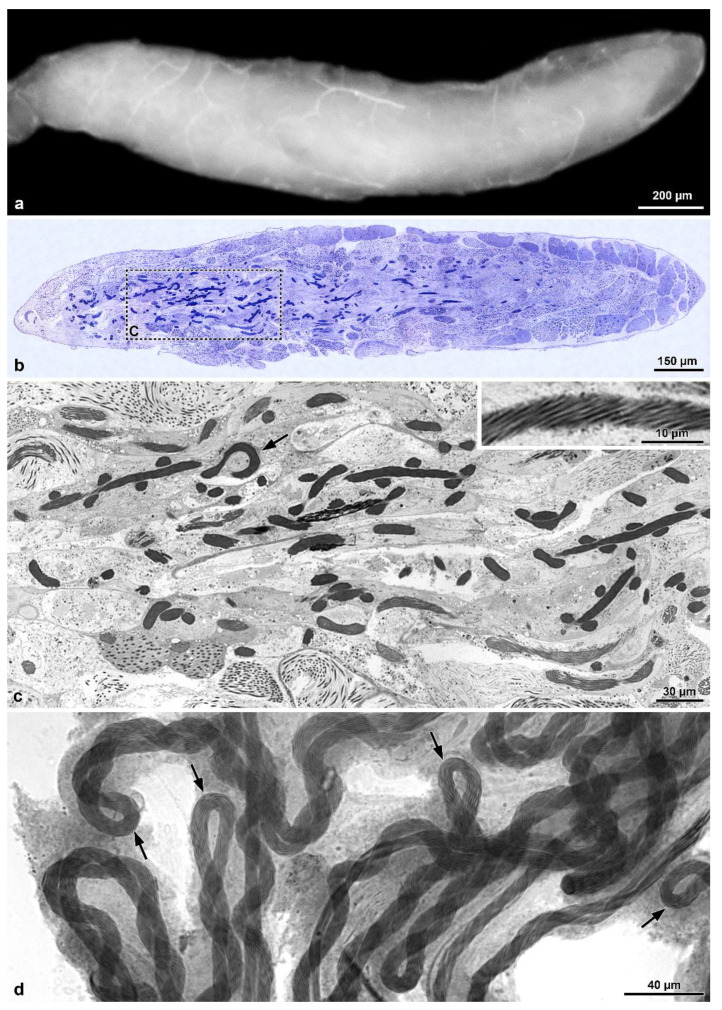
*Hoshihananomia* sp. Light microscopy showing a whole testicular follicle and the folding of the sperm bundles. (**a**,**b**) Testicular follicles entire and in longitudinal section stained with Giemsa. Note the cysts more advanced preferentially arranged along the central region. (**c**) Higher magnification of the dashed region in the previous figure. Note that the cysts are folded (arrow), their two portions coiled over each other, and in addition, the sperm are coiled in the cyst (**inset**), so that the whole set forms a superhelix. (**d**) Testicular cysts in advanced stage stained with acetic orcein. Note all the cysts are folded at about halfway down their (arrow) length and the two halves wrapping around on each other.

The reproductive system anatomy of *Hoshihananomia* sp. is very similar to that of the previous species. Five follicles form each of the two testes; however, their follicles are elongated (400 × 2100 µm, Figure 5a,b) rather than oval. Each follicle is filled with cysts at different stages of spermatogenesis, with the youngest cysts distributed in the anterior and peripheral regions of the follicle.

**Figure 6:**
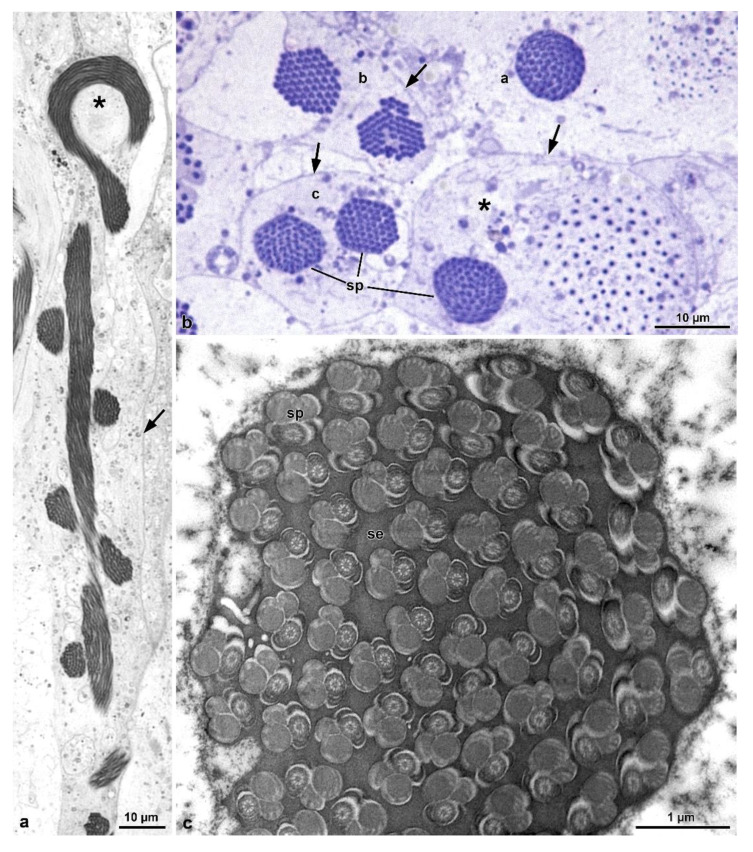
*Hoshihananomia* sp. (**a**) The TEM micrographs illustrate the fine structure of flagella at different levels. Detail of one sperm cyst to show the looping point (asterisk) and the plasma membrane of the cyst cell (arrow). (**b**) Cross-section of the sperm cysts showing the looping of the sperm bundles (sp); two sperm cysts are sectioned through the central flagellar region (a–c) while that on the bottom right (asterisk) is crossed along the flagellar posterior ends, where the sperm flagella become narrowed and stiff. Arrows indicate the plasma membrane of the cyst cells. (**c**) TEM of a cross-sectional cyst showing sperm (sp) embedded in dense and homogeneous material (se).

**Figure 7:**
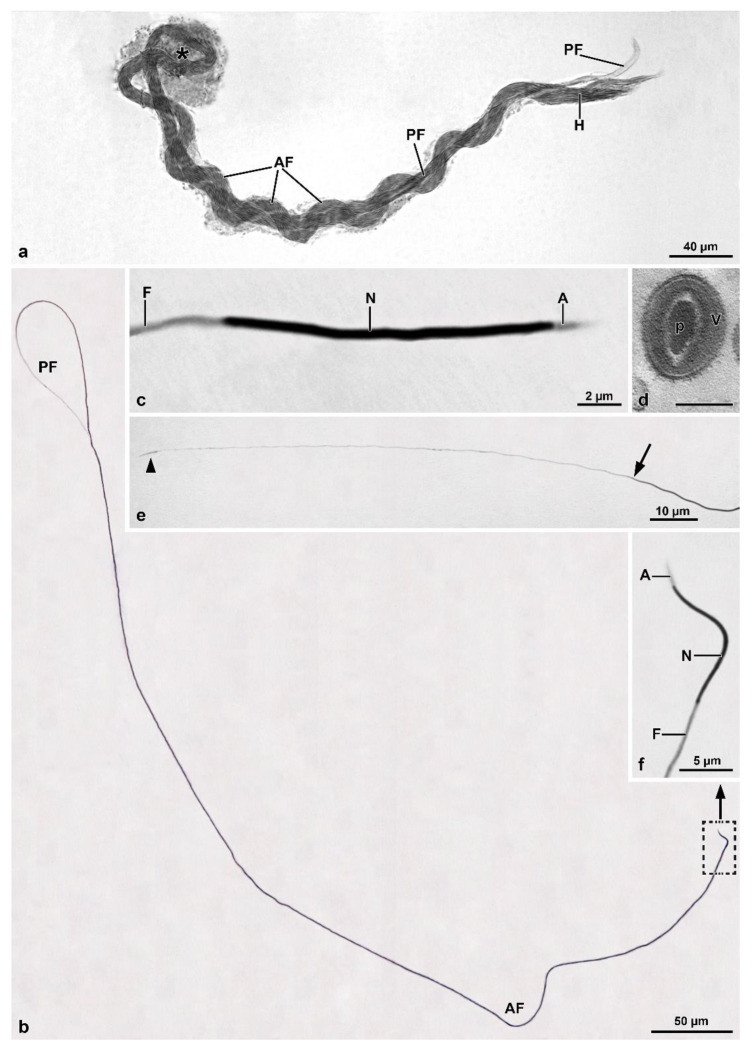
*Hoshihananomia* sp. Light microscopy of a whole sperm and of a sperm cyst showing the sperm looping. (**a**) An isolated sperm cyst showing the sperm looping at half-length (asterisk), its end with the sperm heads (H) and the stiff posterior regions (PF). AF, anterior flagellar region. (**b**,**f**) A whole sperm showing the head with acrosome (A), nucleus (N), and flagellum (F). PF, posterior stiff flagellum. (**c**,**d**) Detail of the head region with the acrosome (A), the nucleus (N), and the beginning of flagellum (F); p, perforatorium; V, acrosomal vesicle. (**e**) Detail of the posterior stiff flagellar region. Arrow indicates the beginning of the stiff region; and arrowhead the posterior tip.

In contrast, cysts in more advanced stages of maturation are observed throughout almost the entire central region (Figure 6b,c). During spermiogenesis, as the cysts elongate, the sperm bundle spiral (Figure 5c, inset) and fold at their median region, forming a loop approximately 460 µm away from the region of the sperm heads (Figure 5d, Figure 6a and Figure 7a). The loop faces the distal region of the follicle, while the two cystic ends are directed towards the efferent duct (Figure 5b,c). Finally, the two halves of each cyst coil over one another so that the whole forms a double helical structure, which is easily observed in cysts (Figure 5d and Figure 7a), sectioned longitudinally (Figure 6a).

**Figure 8:**
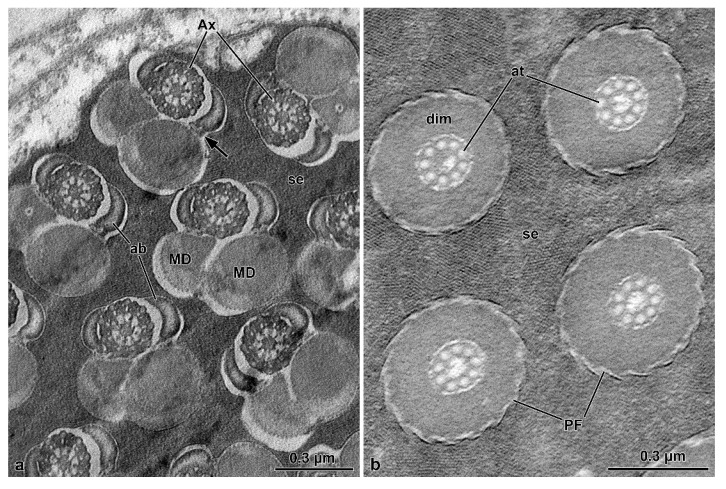
*Hoshihananomia* sp. TEM micrographs showing the sperm flagella at different levels. (**a**) A small portion of a cyst showing sperm flagella displaying an axoneme (Ax), two asymmetric mitochondrial derivatives (MD), two long, curved accessory bodies (ab), and discrete structures connecting the derivatives to their accessory bodies (arrow). Note the dense material (se) between the flagella. (**b**) Cross-section of the posterior rigid flagellar region (PF). Note that there are only the 9 axonemal accessory tubules (at) embedded in a thick and dense intracellular material (dim). Also note that the flagellar ends are embedded in uniform and also quite dense extracellular material (se).

In isolated cysts, it was possible to observe that all sperm nuclei are positioned at the same end of the cyst (Figure 7a). Cross-sections showed that each cyst contains up to 64 spermatozoa (Figure 6c), embedded in dense material (Figure 8a,b). The total length of the mature cyst (or sperm bundle) is around 1030 µm, with the region after the loop measuring about 570 µm. At approximately 145 µm before the posterior tip of the cyst, a rapid tapering occurs so that the sperm bundle diameter decreases from 8.5 µm to 4.8 µm, and then to 3.0 µm (Figure 5d, Figure 6a and Figure 7a).

In the vas deferens and seminal vesicles, only individualized sperm were observed, indicating that the sperm bundles were dissociated before or immediately downstream of the testes. In this mordellid, the mature sperm are thin and very long, measuring around 1200 µm in length (Figure 7b). The head region comprises the acrosome and nucleus (Figure 7c,d,f), with an average length of 2.5 µm and 16.3 µm, respectively. Posteriorly, as seen in the bundles, the flagellum shows a long (~145 µm) thinner portion, with the tip (~4 µm) that thickens slightly and exhibits an arrowhead shape (Figure 7b,e). The mature sperm exhibits an apical bi-layered acrosome that consists of an elliptical acrosomal vesicle with a compact and slightly flattened perforatorium (Figure 7d). The nucleus-flagellum transition region is similar to that of the previous species, including anterior projection of the centriole adjunct along with the entire nucleus.

The flagellum (Figure 8a) has an axoneme with 9 + 9 + 2 microtubules and a typical intertubular material adherent to the accessory tubules. Two accessory bodies flank the axoneme for most of the flagellar length. They are long and curved in cross-section and have the same size (Figure 6c and Figure 8a). Mitochondrial derivatives, in cross-section, are asymmetrical in size and shape; the thicker is almost circular, located in an opposed position to the axoneme, and with paracrystalline material occupying more than half of its diameter. The thinnest one has a drop-shaped appearance, and the paracrystalline material occupies a large part of its pointed region (Figure 6c and Figure 8a). Between the mitochondrial derivatives and the accessory bodies, there is a discrete dense material connecting these structures, like in other tenebrionoids (Figure 8a). The tail end is characterized by the disappearance of the accessory bodies, mitochondrial derivatives, and the axonemal microtubule doublets, whereas the accessory tubules persist (Figure 8a). In this region, a thick (0.16 µm) and dense intracellular material surround the nine accessory tubules (Figure 8a), while the flagellar tips are embedded in a dense amorphous extracellular material (Figure 6a and Figure 8b).

### 3.3. Oedemera Nobilis (Oedemeridae)

**Figure 9:**
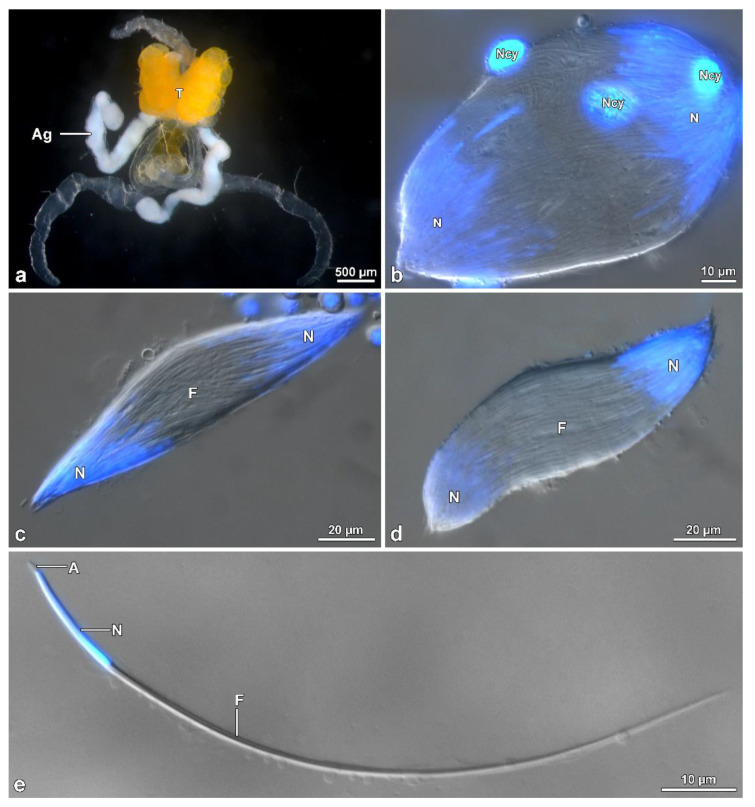
*Oedemera nobilis*. Light microscopy of the male genital system organization and of the sperm cysts at the end of the mitotic divisions, Hoechst stained. (**a**) Male reproductive system with testes (T) and accessory glands (Ag). (**b**) Large sperm cyst after Hoechst staining. The nuclei of the germ cyst (Ncy) and the sperm nuclei (N) are visible. These latter are located at the two opposite poles of the cyst. (**c**,**d**) Two spindle-shaped sperm cysts after Hoechst staining. (**e**) An isolated sperm with acrosome (A), nucleus (N), and flagellum (F) after Hoechst staining.

**Figure 10:**
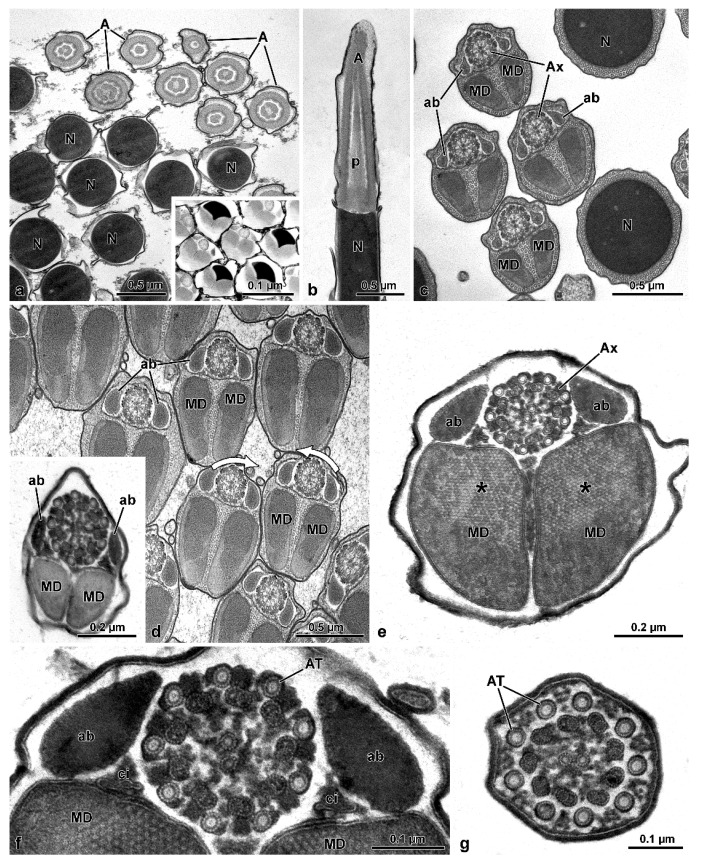
*Oedemera nobilis*. TEM micrographs showing the sperm flagella at different levels. (**a**) Cross-section through the anterior sperm region with the bi-layered acrosome (A), and the cylindrical nuclei (N). The inset shows the transition region nucleus-flagellum with the reduced size of the nucleus. (**b**) Longitudinal section of the apical sperm region showing the acrosome (A) with the perforatorium (p) and nucleus (N). (**c**) Cross-section through the almost mature spermatids with nuclei (N) and the flagella showing the axoneme (Ax), accessory bodies (ab), and mitochondrial derivatives (MD). (**d**) Cross-section of mature spermatids with well-developed accessory bodies (ab), and elongated mitochondrial derivatives (MD). The inset shows a flagellar posterior region, in which the diameters of the accessory bodies and mitochondrial derivatives are greatly reduced. White arrows indicate the orientation of dynein arms. (**e**) Cross-section of the flagellum of a mature sperm with the axoneme (Ax), accessory bodies (ab), and quite similar mitochondrial derivatives (MD) with a large part of the matrix crystallized (asterisks). (**f**) High magnification of the previous figure showing accessory tubules (AT) with 16 protofilaments in their wall, accessory bodies (ab), flattened cisterns (ci) beneath them, and mitochondrial derivatives (MD). (**g**) Cross-section of the tail end with only microtubule doublets devoid of dynein arms, and accessory tubules (AT). Note the reduced amount of the intertubular material.

In this species, the male genital system (Figure 9a) consists of two large yellow-pigmented testes, which continue with large deferent ducts flowing into a long transparent ejaculatory duct. Two long, whitish accessory glands are also present. Testes contain several elliptical follicles filled with elongated germ cysts. During spermiogenesis, the numerous spermatids within a cyst (about 512 as a result of 9 cycles of cell divisions of the initial spermatogonium) are distributed in two groups in opposite directions, each consisting of up to 256 cells. At maturity, sperm cysts are fusiform with sperm nuclei clustered at two opposite poles, while flagella occupy the central region between these poles (Figure 9b–d). The consequence of this sperm organization is a common observation, in cross-sections, of sperm flagella with opposite orientations of their axonemes, deducible by clock-wise or anti-clockwise orientation of dynein arms on microtubule doublets (Figure 10d).

The sperm is relatively short, about 105–110 µm long, with an acrosome, about 2.0 µm long, and a nucleus 16 µm long (Figure 9e and Figure 10b). The conical acrosome has a bi-layered structure; it is 0.4 µm in diameter and has a dense perforatorium that forms an inner ring in cross-section (Figure 10a,b). The cylindrical nucleus has a diameter of 0.56 µm and contains a compact chromatin (Figure 10a–c). It reduces its diameter in the posterior tip and forms two grooves hosting the two mitochondrial derivatives (Figure 10a, inset). The flagellum consists of two large, elliptical, equally developed mitochondrial derivatives (0.71 × 0.25 µm). Its region facing the axoneme shows that a good portion of its matrix is crystallized (Figure 10d,e). Two elliptical accessory bodies with a pointed region opposite to the mitochondrial derivatives are located lateral to the axoneme; their cortical region has a fine structure (Figure 10c–f). The axoneme of 9 + 9 + 2 microtubules shows accessory tubules with 16 protofilaments in their tubular wall (Figure 10f,g). A small cistern surrounded by electron-dense material is present between each accessory body and the corresponding mitochondrial derivative (Figure 6f). Towards the posterior flagellar region, the accessory bodies narrow progressively and the mitochondrial derivatives greatly reduce their size (Figure 10d inset). In the flagellar end (Figure 10g), the two mitochondrial derivatives and the accessory bodies are lost. In the axoneme, accessory tubules are somewhat distant from the microtubule doublets, which lack the dynein arms. Also, in this region, the intertubular material is either very scarce or absent.

### 3.4. Accanthopus Velikensis (Tenebrionidae)

**Figure 11:**
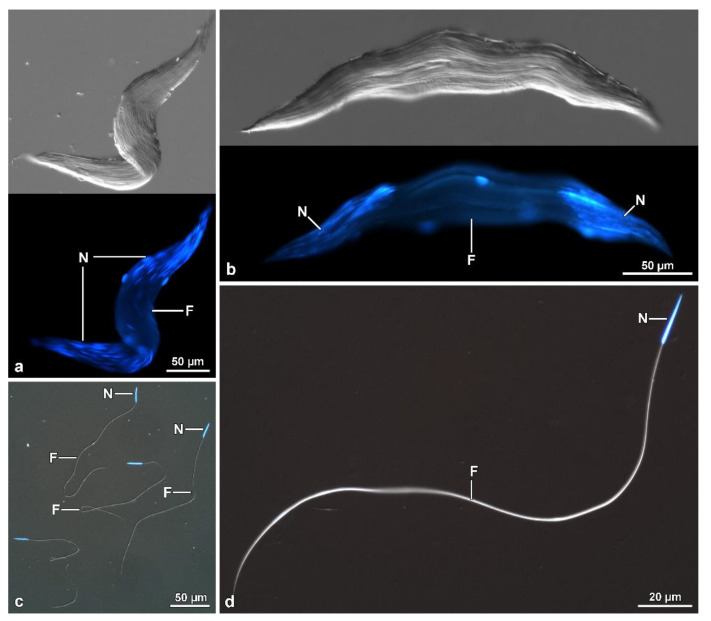
*Accanthopus velikensis.* Light microscopy of the sperm cysts at the end of mitotic divisions, Hoechst stained. (**a**,**b**) Sinuous spindle-shaped sperm cysts at light interference contrast (upper) and after Hoechst staining (bottom) showing the position of sperm nuclei (N) at the two opposite poles, and the flagella (F) along the central region. (**c**,**d**) Free sperm after Hoechst staining with nuclei (N) and flagella (F).

**Figure 12:**
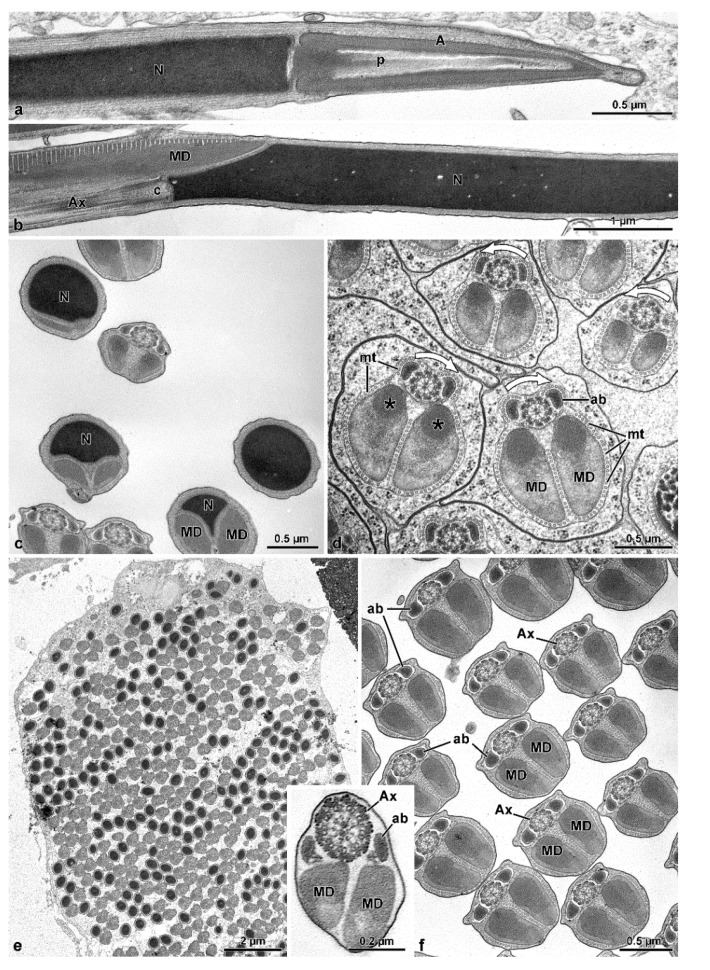
*Accanthopus velikensis.* TEM micrographs illustrating the sperm structure at different levels. (**a**) Longitudinal section of the sperm head with the conical acrosome (A) and the inner perforatorium (p) in front of the nucleus (N). (**b**) Longitudinal section of the posterior nuclear end that has a beak shape and hosts, on one side, the centriole (c) that gives rise to the axoneme (Ax), while on the opposite side the mitochondrial derivatives (MD) are positioned. (**c**) Cross-section of the nucleus-flagellum transition showing nuclei (N) with reduced diameter to host the mitochondrial derivatives (MD). (**d**) Cross-section of almost mature spermatids with the accessory bodies (ab), and the quite similar mitochondrial derivatives (MD), asterisks indicate the crystallization of the matrix. At this stage, many microtubules (mt) surround the structures. Note the opposite orientation of the dynein arms in the axonemes of some sperm (white arrows), indicating the antiparallel arrangement of sperm in the same cyst, as shown after Hoechst staining. (**e**) Cross-section of a cyst showing the numerous sperm cut either in the nuclear or flagellar region, indicating sperm coming from opposite poles. (**f**) Cross-section of almost mature spermatids with axonemes (Ax), mitochondrial derivatives (MD), and accessory bodies (ab). In the inset the flagellum of a mature sperm showing the same structures.

The male reproductive system of this species has two large testes with follicles filled with elliptical germ cysts, 285–300 µm long (Figure 11a–d). Fluorescent dye staining shows that in the cysts, the sperm are distributed in two groups with their nucleus positioned at the two extremities of the cysts, while the flagella extend towards their median region (Figure 11a,b). A cross-section through the cyst reveals that it contains up to 512 germ cells, corresponding to nine cycles of cell divisions of the initial spermatogonium (Figure 12e). Moreover, a cross-section through the middle region of the cyst confirms that sperm cells have axonemes with either clockwise or anti-clockwise dynein arms orientation (Figure 12d), indicative of an opposite sperm orientation.

The sperm is about 240–250 µm long (Figure 11e,f). Apically, it shows a 2.2 µm long conical acrosome with a dense perforatorium (Figure 12a). The 20 µm long nucleus (Figure 11f), is cylindrical, with a constant 0.6 µm diameter (Figure 12a) for all its length till its posterior end (Figure 12b,c). The posterior nuclear region is adapted to host the two mitochondrial derivatives, thus becoming progressively narrowed (Figure 12c). Immediately below the nucleus and opposite to the mitochondrial derivatives, the centriole region is evident, from which the axoneme begins (Figure 12b). The latter consists of a 9 + 9 + 2 microtubule complex flanked by two triangular accessory bodies (Figure 12d,f). Two mitochondrial derivatives with similar size (0.65 × 0.30 µm) and their region facing the axoneme crystallized are visible (Figure 12d). At the tail end, only the axoneme with doublets devoid of dynein arms is still visible, with the two mitochondrial derivatives and, more distally, the accessory bodies disappearing.

## 4. Discussion

The sperm ultrastructure of the four species studied here not only confirms our previously published data (see [9]) but, in addition, provides new useful data for a better understanding of the phylogenetic relationships between the various families of Tenebrionoidea. In Oedemeridae, the new family examined here, spermatozoa show the same organization seen in the other advanced members of the superfamily [7,8,9,11]. They consist of a short two-layered acrosome, relatively long nucleus, and flagellum; in this latter, an axoneme with 9 + 9 + 2 microtubules, two thick similar mitochondrial derivatives, and two elliptical accessory bodies. However, the features supporting the positioning of this family together with advanced tenebrionoids are the presence of numerous sperm per testicular cyst and the antiparallel arrangement of the spermatozoa within the cyst. These two remarkable features are shared by all tenebrionoid families studied so far, except Mordellidae ([9], this study) and Ripiphoridae [9]. The unusual arrangement of sperm within the cysts was initially described in Tenebrionidae by Dias et al. [8]. These authors demonstrated that this disposition begins in the first stages of spermiogenesis; as the flagella elongate, half of the nuclei migrate to one pole of the cyst and the other half to the opposite pole, ultimately forming two antiparallel sets of sperm per cyst, which are easily observed using DNA stains. Observation by transmission electron microscopy further demonstrated that this arrangement could be inferred from the clockwise and counter-clockwise orientations of axonemal microtubule pairs in cross-sections of cysts (see [9]) and that this character is common to most Tenebrionoidea. The amount of sperm per cyst is a consequence of the number of division cycles that the initial spermatogonium undergoes in the early stages of spermatogenesis, a number considered constant for the species. In four species of Tenebrionidae [8], as well as in *A. velikensis* and *O. nobilis* of this study, up to 512 (=2^9^) sperm per cyst were observed, indicative of nine spermatogonial division cycles. On the other hand, in the Tenebrionidae *Lagria villosa* (Fabricius, 1781), 1024 (=2^10^) sperm were counted per cyst [15]. Although Zhang et al. [5] suggested that Ciidae was closely related to the Tenebrionidae, in *Ceracis cornifer* (Melli, 1849), 256 sperm per cyst were observed [11], and the same number was found in four species of Meloidae [9]. In families of Tenebrionoidea that are considered basal, such as Ripiphoridae and Mordellidae, the number of sperm per cyst is lower, reaching 64 (=2^6^) ([9], this study). Commonly, the works on Oedemeridae, Pythidae, Meloidae, Ciidae, and Tenebrionidae placed these groups in all the main branches of the phylogenetic trees above the more basal branch of Mordellidae and Ripiphoridae [4,5,6,15,16]. Thus, it is possible to suppose that the antiparallel disposition of the sperm inside the cyst, and the high number of them per cyst, are characteristics that arose in the common ancestor of all Tenebrionoidea, except Mordellidae and Ripiphoridae. Yet these data indicate that the proposition by Virkki [17] and Lachaise and Joly [18] that the occurrence of a relatively low number of sperm per cyst would indicate a derived character state, compared to cysts containing a relatively high number, cannot be applied to all Tenebrionoidea, as recent studies have indicated that Ripiphoridae and Mordellidae would rather form a sister group to all other Tenebrionoidea [5,6].

Within the Mordellidae family, *Mordellistena* sp. [9] and *Hoshihananomia* sp. have giant sperm, 1230 µm and 1030 µm long, respectively, while those of *M. brevicauda*, with about 290 µm, are comparatively very short. Based on molecular data, Batelka et al. [19] showed, in one of the cladograms, the genera *Mordellistena* and *Hoshihananomia* in the most basal and most derived branches, respectively. Thus, it is possible to assume that giant sperm may be the synapomorphic condition for Mordellidae. In contrast, short sperm, as observed in *M. brevicauda*, is a derived condition within the family. Among all the tenebrionoids studied so far, giant spermatozoa were observed only in Mordellidae and in Ripiphoridae ([9,20], this study), which is consistent with the proposal that both families are closely related and form a sister group of all Tenebrionoidea [5,6].

A new peculiar finding observed in the two Mordellidae species studied here and possibly in *Mordellistena* sp. and the Ripiphoridae *Ptilophorus dufourii* [9], deserves to be discussed. The sperm bundles in these species are characterized by bending at half their length forming a loop at this point. In *Hoshihananomia* sp., probably because the sperm are very long, it is easy to see that the two folded halves spiral over each other. In *M. brevicauda*, this cystic organization is less evident, as the sperm length is only about one-quarter of that in H. sp. However, a twisting of the median region of the sperm bundle is also evident in this species. Thus, the cyst shows in both species a supercoiled organization. The folding of the sperm bundle forming a loop, the spiraling of the two halves, and the twisting of the sperm are probably spermatogenic mechanisms resulting from evolutionary innovations that enabled males with extremely long sperm to produce them in sufficient quantities to be competitive, even in relatively small testes. It was possible to observe that the folding of the sperm bundle within a cyst in the form of a loop begins in the early stages of spermiogenesis, and according to Syed et al. [13], it is the result of asynchrony between the elongation of spermatid tails and increases in the plasma membrane of surrounding somatic cells. It is essential to point out that this peculiar cystic organization occurs in mordellids either with very long sperm, namely *Hoshihananomia* sp. and *Mordellistena* sp. [9], as well as with very short sperm, such as *M. brevicauda*. Therefore, this feature must be common to the entire family and it may be likely present in the common ancestor of Mordellidae and Ripiphoridae, sister taxa sharing this cystic organization.

Testicular follicles in *H*. sp. are elongated and approximately four times longer than those of *M. brevicauda*, which are ovoid. In tenebrionids the testicular follicles are oval, with the cysts in the different stages distributed in distinct transverse zones: the youngest in the distal zone and the most advanced (about 100 µm long) in the proximal zone to the efferent duct (unpublished data). Such a feature is different from what was observed in *Hoshihananomia* sp., in which the long cysts are distributed along almost the entire central region of the follicles. These differences seem to validate an existing correlation between sperm and testis lengths and the organization of cysts within follicles.

Mordellidae sperm ([9], this study) are characterized by a long posterior flagellar tip. In many insects this region is affected by axoneme disorganization with the microtubule doublets becoming grossly irregular structures and lacking their dynein arms, as described in the orthopteran *Gryllotalpa gryllotalpa* [10,21], the zorapteran *Zorotypus caudelli* [10], and in the dipterans *Drosophila melanogaster* and *Bactrocera oleae* [10]. In the mordellids, however, axonemal degeneration occurs by the loss of the central 9 + 2 microtubule complex, while accessory tubules persist throughout this entire region. Due to the lack of microtubule pairs and consequently dynein arms, this region is stiff and immotile. Still in Mordellidae ([9], this study), unlike the other Tenebrionoidea, there is a compact material among the sperm in the cysts; in the sperm, the centriole adjunct flanks the entire nucleus, and the tail posterior end is long, formed only by axonemal accessory tubules embedded in dense intracellular material. The absence of these features in ripiphorids, and the phylogenetic distance of *Mordellistena* and *Hoshihananomia* [19], suggest that they may be unique traits (autopomorphies) of Mordellidae. Furthermore, these traits may constitute good phylogenetic signals to understand the relationships between the basal families of Tenebrionoidea. Alternatively, as suggested by Hunt et al. [15], the clade at the base of Tenebrionoidea could be Ripiphorinae and Mordellinae together with two subfamilies of Lymexyloidea.

The sperm flagellar structure of insects is also characterized by the size and shape of the mitochondrial derivatives and the accessory bodies [10]. All the species have their two mitochondrial derivatives with a similar shape. However, these structures are more developed and elliptical in *O. nobilis* and *A. velikensis* while in the two species of Mordellidae they are smaller and oval-shaped. The two accessory bodies, flanking the axoneme, are of primary importance to verify the relationship between the families of Tenebrionoidea. It has been already pointed out that the oval or elliptical shape of these structures in cross-section, is typical of various species of Tenebrionidae such as *Tenebrio molitor* or *Tribolium castaneum* [7,9] and also of *A. velikensis* here studied. This shape, with few variations, is the commonest feature among the whole superfamily, and it was also found in Meloidae [9,20]. *O. nobilis*, a member of the new family here studied has elliptical accessory bodies with a pointed apical side. On the contrary Mordellidae species have smaller accessory bodies with an almost triangular shape ([9], this study).

As quoted in the Introduction, Levkaničová [3] and Bocak et al. [4] considered Ripiphoridae, Mordellidae, and Meloidae closely related. However, the latter authors considered the clade with the three families the most derived, while Levkaničová considered it the most basal. From the testicular and sperm morphology ([9,20], this study) it is possible to assume that Mordellidae and Ripiphoridae, but not Meloidae, share a recent common ancestor, a condition also proposed by Zhang et al. [5] and McKenna et al. [6] from extensive gene sampling. They further suggested that the clade with these two families is at the base of the superfamily. A position that will possibly be supported by sperm and testicular morphology, but this type of data should be extended to other families and also to groups closely related to Tenebrionoidea, such as Lymexyloidea.

## 5. Conclusions

This study confirms that Mordellidae are closely related to Ripiphoridae and that both occupy a phylogenetically distinct position concerning other tenebrionoids. In mordellids, the number of sperm per cyst is low compared to the Oedemeridae and Tenebrionidae, also studied here. Furthermore, in these latter two families, the sperm of the same cyst are distributed in two sets arranged antiparallelly, as in the other families of Tenebrionoidea, except mordellids and ripiphorids. In these latter families, the long sperm cells exhibit the same orientation within each cyst, and the sperm bundle forms a loop approximately at half its length to be contained within a smaller cyst. Also, sperm are characterized by thin mitochondrial derivatives and accessory bodies and, in mordellids, by a long, rigid, immotile posterior flagellar region in which accessory tubules are embedded in dense intracellular material.

## Data Availability

Data are contained within the article.
